# Evaluation of rheumatic causes underlying childhood-onset arthritis

**DOI:** 10.55730/1300-0144.6034

**Published:** 2025-06-14

**Authors:** Seher ŞENER, Erdal ATALAY, Ezgi Deniz BATU, Cansu Ayten TATAR, Özge BAŞARAN, Yelda BİLGİNER, Seza ÖZEN

**Affiliations:** 1 Division of Pediatric Rheumatology, Department of Pediatrics, Faculty of Medicine, Hacettepe University, Ankara, Turkiye; 2 Department of Pediatrics, Faculty of Medicine, Hacettepe University, Ankara, Turkiye

**Keywords:** Arthritis, childhood-onset, rheumatic etiologies

## Abstract

**Background/aim:**

Childhood-onset arthritis may result from various rheumatic diseases. This study aimed to systematically evaluate their distribution and characteristics in a large pediatric cohort.

**Materials and methods:**

We retrospectively analyzed all pediatric patients who presented with arthritis to the pediatric rheumatology clinic between January 2000 and January 2023.

**Results:**

A total of 1713 pediatric patients who presented with arthritis were included in this study (median age at diagnosis: 6.8 years; F/M = 1.2). Most of the patients (n = 859, 50.1%) had juvenile idiopathic arthritis (JIA) (especially oligoarticular JIA). Patients with familial Mediterranean fever, comprising 277 individuals (16.2%) presented with arthritis. The majority of them (n = 203, 73.3%) had short-lasting recurrent oligoarthritis (mostly monoarthritis) attacks. While arthritis was observed in 236 (13.8%) patients with vasculitis, immunoglobulin A vasculitis was the most common cause among them (n = 166, 70.3%). Most of the vasculitis patients had oligoarthritis (n = 221, 93.6%). Reactive arthritis was present in 147 patients (8.6%, mostly monoarthritis). Acute rheumatic fever was another cause of arthritis (4.7%, mostly polyarticular and migratory). Among autoimmune diseases (mostly systemic lupus erythematosus), arthritis was detected in 82 patients (4.8%, oligoarthritis or polyarthritis).

**Conclusion:**

In our study, we focused on the rheumatic etiologies underlying childhood arthritis. Given the heterogeneity of etiologies, clinical evaluation should be comprehensive, considering systemic features beyond joint involvement.

## 1. Introduction

There are several etiologies of childhood arthritis, each with its unique characteristics, which are categorized based on the number of joints affected, the presence of other symptoms, and specific clinical features [1].

The most common rheumatic cause underlying childhood arthritis is juvenile idiopathic arthritis (JIA) [2]. JIA is divided into seven subgroups: Oligoarticular JIA (arthritis in four joints or less), rheumatoid factor (RF) negative or positive polyarticular JIA (arthritis in five or more joints), systemic JIA (arthritis plus systemic symptoms), enthesitis-related arthritis (ERA), psoriatic arthritis (PsA), and undifferentiated arthritis [3]. Besides JIA, a wide variety of autoinflammatory and autoimmune diseases can also cause arthritis [4]. Familial Mediterranean fever (FMF) is one of the leading autoinflammatory causes of arthritis [5,6]. In addition, autoinflammatory periodic fever syndromes other than FMF can cause recurrent episodes of inflammation in joints and other parts of the body [7–9]. Among systemic autoimmune diseases, juvenile systemic lupus erythematosus (SLE) involves various symptoms, including joint inflammation [10]. Although rare, arthritis can also be seen in other autoimmune conditions such as juvenile dermatomyositis (JDM), juvenile scleroderma, mixed connective tissue disease (MCTD), and Rhupus syndrome (coexistence of JIA and SLE) [11]. Another important cause of arthritis is vasculitis [11]. The type of vasculitis in which arthritis is most commonly seen is immunoglobulin A vasculitis (IgAV), which is small vessel vasculitis [12].

Diagnosing the specific etiology of childhood arthritis can be challenging, and it often involves a combination of clinical assessment, laboratory tests, imaging, and, in some cases, genetic analysis [13,14]. Treatment approaches may vary depending on the underlying cause and the patient’s specific symptoms and needs. Early diagnosis and appropriate management are crucial to improving outcomes for children with rheumatic diseases [14].

Understanding the diverse etiologies of childhood arthritis is essential for early diagnosis, appropriate management, and improved outcomes. Therefore, in our study, we aimed to evaluate the underlying rheumatic etiologies in patients presenting with arthritis in childhood.

## 2. Materials and methods

This study was granted ethical approval by our center’s ethics committee (approval number: GO 21/967; date: 07.11.2021). The study was conducted in accordance with the ethical guidelines established in the 1964 Declaration of Helsinki and its subsequent revisions.

### 2.1. Patient population and data collection

All pediatric patients (< 18 years) who presented with arthritis between January 2000 and January 2023 were retrospectively evaluated. Although the study was initiated in 2021 with retrospective ethical approval, patients diagnosed after this date were also included retrospectively based on their completed medical records, without prospective data collection. When diagnosing, the International League of Associations of Rheumatology (ILAR) criteria were used for all JIA patients [3], American College of Rheumatology (ACR) criteria for reactive arthritis patients [15], Eurofever/PRINTO criteria for patients with autoinflammatory periodic fever syndromes [16], modified Jones criteria for patients with acute rheumatic fever (ARF) [17], the Systemic Lupus International Collaborating Clinics (SLICC) classification criteria for SLE patients [18], Bohan and Peter criteria for JDM patients, Kasukawa [19,20], Alarcon–Segovia and Villareal criteria for patients with MCTD [21], Ankara 2008 (EULAR/PRES/PRINTO) criteria for patients with IgAV or polyarteritis nodosa (PAN) [22,23], the 2015 international pediatric Behcet’s disease criteria (PEDBD) for patients with Behcet’s disease [24], and the American Heart Association (AHA) criteria for patients with Kawasaki disease [25]. Patients with rhupus syndrome met both ILAR and SLICC criteria [3,18]. Patients with toxic synovitis had transient synovitis of the hip joint. Chronic nonbacterial osteomyelitis (CNO) patients had clinical symptoms lasting > 6 weeks with unifocal or multifocal inflammatory bone lesions [26]. All patients with adenosine deaminase 2 (*ADA2*) deficiency had homozygous *ADA2* gene mutations. All diagnoses were confirmed by board-certified pediatric rheumatologists using standardized criteria for each condition. Ambiguous cases were reviewed by senior clinicians to ensure consistency across the study period. A titer of > 1/100 was considered positive for antinuclear antibody (ANA).

Patients with nonrheumatic etiologies as the cause of arthritis were excluded. In addition, septic arthritis was excluded in patients with toxic synovitis, and infections and malignancies were excluded in patients with CNO.

All patients’ age at diagnosis, sex, etiologies of arthritis, clinic and laboratory findings, disease duration, and outcomes were evaluated. Outcomes were divided into two categories: “improvement” and “no improvement”. Improvement was defined for all patients as being a clinically asymptomatic disease (absence of arthritis and other disease-related findings), having negative acute phase reactants (erythrocyte sedimentation rate [ESR] and C-reactive protein [CRP]), and having no active findings on recent musculoskeletal system radiological imaging (if present). Improvement status was assessed at the most recent follow-up, and only patients with a minimum of 6 months of follow-up were included in the outcome analysis.

### 2.2. Statistical analysis

We utilized IBM SPSS software version 25.0 (IBM Corp., Armonk, NY, USA), to analyze all the data. Descriptive statistics were expressed in terms of frequency (n) and percentage (%), median [1st–3rd quartiles (Q1–Q3)]. To assess the normal distribution of numeric variables, we employed both visual examination and analytical methods (Shapiro–Wilk’s test). In cases where clinical or laboratory data were missing, those specific variables were excluded from subgroup analyses. However, patients were not excluded from the overall cohort as long as the diagnosis of a rheumatic disease was confirmed.

## 3. Results

During the study period, 1921 pediatric patients presented with arthritis. After excluding 208 patients with nonrheumatologic causes, a total of 1713 patients with confirmed rheumatic diseases were included in the study. Among these 1713 included patients, 94.1% had arthritis at presentation, while 5.9% developed arthritis during follow-up (Tables 1 and 2). The median age of patients at diagnosis was 6.8 (4.7–12.5) years, and 940 (54.9%) were female. Detailed arthritis features of different rheumatologic etiologies were summarized in Table 3. In addition, a summary comparison of the most common etiologies is presented in Table 4.

JIA was the leading diagnosis (50.1%), with the oligoarticular subtype being most frequent (46.1%) (Figure). JIA patients presented with a chronic and persistent disease course requiring long-term management. Comorbidities such as uveitis and inflammatory bowel disease (IBD) were most frequently present in patients with oligoarticular JIA subtype. ANA positivity in other JIA subtypes and human leukocyte antigen (HLA) B27 positivity in ERA were important findings, except systemic JIA. Although the arthritis features of the patients were variable, a significant number of patients with ERA also had sacroiliitis (n = 173, 88.7%) or enthesitis (n = 43, 21.9%) as musculoskeletal findings other than arthritis. Improvement was observed in the majority of JIA patients (94.4%), with the highest rate seen in those with oligoarticular JIA (98.7%).

Patients with FMF, comprising 277 individuals (16.2%), presented with arthritis. The majority of patients (n = 203, 73.3%) had oligoarthritis (mostly monoarthritis), and arthritis typically occurred during FMF attacks. All patients had elevated acute phase reactants during arthritis. Arthritis was also present in two patients with cryopyrin-associated periodic syndrome (CAPS) and one patient with mevalonate kinase deficiency (MKD). In contrast to JIA, FMF-associated arthritis was short-lived and recurrent, often resolving spontaneously within days and rarely causing joint damage.

While arthritis was observed in 236 (13.8%) vasculitis patients, IgAV was the most common cause among them (n = 166, 70.3%). Most patients had oligoarthritis (n = 221, 93.6%). All patients had accompanying systemic findings and most of them had high acute phase reactants (n = 201, 85.2%). Although arthritis was common in IgAV, it was typically mild and transient, and was accompanied by systemic features that distinguished it from JIA.

Reactive arthritis was present in 147 patients (8.6%). Most of these patients (n = 126, 85.7%) had a history of upper respiratory tract, gastrointestinal, or genitourinary system infections 1–2 weeks before the arthritis. Additionally, a large portion (n = 129, 87.8%) presented with monoarthritis. Reactive arthritis differed from autoimmune forms in its strong temporal link to preceding infections and its generally self-limited nature.

Acute rheumatic fever was another important cause of arthritis (n = 81, 4.7%). A significant number of patients had carditis or other additional disease-specific criteria. Arthritis was mostly polyarticular (n = 58, 71.6%). Unlike other forms, arthritis in acute rheumatic fever was migratory (71.6%) and closely associated with evidence of recent streptococcal infection (100%).

Among autoimmune diseases, arthritis was detected in 82 patients (4.8%) with SLE, JDM, MCTD, rhupus syndrome, and localized scleroderma. Arthritis was seen at similar rates in the oligoarthritis form (n = 41, 50%) and the polyarthritis form (n = 41, 50%). ANA positivity was quite common (n = 72, 87.8%) in this group of patients. Compared to JIA and FMF, autoimmune disease-related arthritis often coexisted with systemic organ involvement and serologic abnormalities.

Among the rarer causes of arthritis were toxic synovitis in 12 patients, CNO in 12 patients, Blau syndrome in two patients, and type 1 interferonopathies in two patients (one with Aicardi–Goutières syndrome and the other with STING-associated vasculopathy with onset in infancy). Such rare genetic and systemic inflammatory disorders have been implicated when arthritis presents atypically or is unresponsive to conventional treatments.

## 4. Discussion

This study provides a comprehensive evaluation of the rheumatic causes of childhood arthritis over a 23-year period, highlighting both common and region-specific etiologies. Our findings reveal not only the dominance of JIA, but also the relatively high prevalence of conditions such as FMF, vasculitis, reactive arthritis, autoimmune diseases, and ARF.

Juvenile idiopathic arthritis is the most common reason for chronic arthritis [27]. It is typically diagnosed when a child has persistent arthritis in one or more joints lasting for more than 6 weeks, and this occurs before the age of 16 years [13]. There are many studies in the literature examining the characteristics of JIA patients. In a large cohort study, Hyrich et al. [28] evaluated 740 JIA patients. Their median age at presentation was 7.6 years (64% girls). The most common ILAR subtype was oligoarthritis (48%) as in our study. In another study evaluating 434 JIA patients, the distribution among the JIA categories was 3.2% systemic, 27.4% persistent oligoarticular, 19.6% extended oligoarticular, 16.4% RF negative polyarticular, 1.4% RF positive polyarticular, 6.5% psoriatic, 10.4% ERA, and 15.2% undifferentiated JIA [29].

Arthritis is one of the common findings of FMF, and sometimes it remains the only major manifestation of the disorder [6]. Although the most common type of arthritis in FMF typically manifests as recurrent, self-limited, and short-lasting acute joint inflammation, chronic forms with protracted joint effusion have been reported occasionally [30]. In a retrospective study, 124 FMF patients with arthritis were investigated [30]. The mean age at the onset of disease was 5.93 ± 3.50 years. Arthritis was recurrent in 106 (85%) patients. All acute findings related to arthritis resolved in 1 week in most patients. None of the patients experienced irreversible joint damage. In this study, arthritis recurred in attacks and resolved in a short time in patients similar to our study.

One of the important conditions in which arthritis is frequently seen is vasculitis [11,31]. Especially in IgAV, arthritis is one of the common clinical findings that can be seen at a rate of 70%–90% [32]. Liao et al. [33] retrospectively reviewed 484 pediatric patients with IgAV. Arthritis/arthralgia was detected in 361 patients (74.6%), and it was found more frequently in younger children (≤ 6 years; p < 0.001). In another study, it was reported that 112 (55.7%) of 201 IgAV patients had arthritis/arthralgia [34]. Although rarer, Behcet’s disease and Kawasaki disease are also important vasculitides that may cause arthritis [35, 36]. In a study evaluating 110 pediatric Behcet’s disease patients, arthritis was detected in 25 patients (22.7%) (14 oligoarthritis, eight polyarthritis, and three monoarthritis) [36]. In another study evaluating 414 pediatric patients with Kawasaki disease, the prevalence of arthritis was found to be 7.5% (n = 31) [35].

When comparing JIA, FMF, and IgAV, which were the three most common causes of arthritis in our cohort, JIA typically presents as chronic joint inflammation requiring long-term immunosuppressive treatment, whereas FMF and IgAV are associated with short-duration, self-limiting episodes of arthritis. Unlike JIA, these latter conditions rarely result in permanent joint damage or deformity. In contrast to FMF, where arthritis tends to be episodic, JIA follows a chronic and persistent course.

Reactive arthritis and acute rheumatic fever represent infection-related arthritis with distinct features. Both conditions respond well to treatment and show no long-term joint damage. Generally, reactive arthritis is presented as an oligoarthritis following infection in the respiratory, genitourinary, or gastrointestinal tracts [37]. In addition, it differs from autoimmune forms in its strong temporal link to preceding infections and its generally self-limited nature. In a systematic review, a total of 44 pediatric patients with reactive arthritis were evaluated [38]. Of these patients, 14 (31.8%) were female, and their ages ranged from 6 months to 14.3 years. The study analyzed the previous infection histories of the patients, revealing that 23 patients (52.3%) had symptoms of upper respiratory tract infection, three patients (6.8%) had pneumonia, and nine patients (20.5%) exhibited abdominal symptoms. In our study, most of the patients had a history of infection [38]. Acute rheumatic fever is an inflammatory condition that emerges several weeks following a throat infection primarily triggered by group A β-hemolytic streptococci [39]. In ARF, arthritis is usually migratory and polyarticular [40]. Arthritis is often accompanied by various additional findings, especially carditis [39]. In their study where Güneş et al. [41] evaluated 166 ARF patients, 127 patients (76.5%) had arthritis. Most had polyarthritis (54.2%), and the majority of patients (94.6%) also had a history of carditis.

Arthritis is a common condition in autoimmune diseases, especially SLE, and is included in the diagnostic criteria [18]. Compared to JIA and FMF, autoimmune disease-related arthritis often coexists with systemic organ involvement and serologic abnormalities, complicating diagnosis and treatment. In a study conducted in 2015 on a large childhood-onset SLE cohort, 32 of 852 patients (3.7%) had arthritis [42]. Chronic monoarthritis was observed in four SLE patients, oligoarthritis in nine, and polyarthritis in 19. Six patients had rhupus syndrome. Rhupus syndrome should also be considered in SLE patients, especially if chronic and/or erosive arthritis is present, unlike classical SLE arthritis [43].

Our findings show consistency with international data, particularly regarding JIA, which remains the most common diagnosis in pediatric arthritis worldwide. However, a notable difference is the higher prevalence of FMF and IgAV in our cohort compared to large-scale cohorts from North America and Europe. The increased prevalence of FMF and IgAV in our cohort is likely due to genetic and regional factors. FMF is caused by mutations in the *MEFV* gene, which is highly prevalent in Mediterranean populations. Studies such as those by Lidar et al. [6] and Ozdogan et al. [30] have shown that the frequency of FMF-related arthritis is significantly higher in Turkey compared to North American or European cohorts. Similarly, IgAV is more frequently observed in Mediterranean and Asian populations [33], which may explain its high prevalence in our cohort.

In resource-limited centers/countries, our findings suggest that clinicians should prioritize diagnosing conditions such as FMF, IgAV, reactive arthritis, ARF, and autoimmune diseases (e.g., SLE, JDM) that can present with clinical features similar to more common forms of arthritis, such as JIA. By considering regional genetic factors and prevalent diseases, physicians can use clinical history and simple laboratory tests (e.g., acute phase reactants, family history) to guide their initial diagnostic approach. For example, in regions where FMF is endemic, the presence of recurrent monoarthritis and fever, with elevated acute phase reactants, should raise suspicion for FMF. Early initiation of colchicine therapy may be considered. Similarly, IgAV should be suspected in children presenting with arthritis and palpable purpura, in regions where this disease is endemic. In areas where reactive arthritis is more common (e.g., after gastrointestinal or urogenital infections), clinicians should consider it in children presenting with a history of infections followed by arthritis, particularly monoarthritis or oligoarthritis. For ARF, which remains a leading cause of arthritis in regions with high rates of streptococcal throat infections, the classic presentation of migratory polyarthritis with a history of a recent sore throat or skin rash should prompt consideration of ARF. Early treatment with penicillin and antiinflammatory therapy should be initiated promptly to prevent long-term cardiac complications. In regions where autoimmune diseases such as SLE and JDM are more common, the presence of typical systemic symptoms (e.g., butterfly rash, photosensitivity in SLE) or proximal muscle weakness (in JDM) should raise suspicion. Early screening for these diseases can improve the prognosis by initiating immunosuppressive therapies early in the disease course. These insights can help streamline diagnosis and avoid unnecessary diagnostic testing in resource-constrained environments. By considering regional epidemiology, clinical presentation, and simple laboratory tests, clinicians can make more efficient and accurate diagnoses, leading to more timely and appropriate management.

This study has several limitations that should be acknowledged. First, due to its retrospective design, some clinical and radiological data were missing, and not all patients had long-term follow-up. Especially, posttreatment radiological evaluations were unavailable for most patients, limiting the assessment of structural joint outcomes. Second, the study covers a long period of 23 years (2000–2023), during which diagnostic criteria and classification systems for various rheumatic diseases have evolved significantly. These changes may have influenced diagnostic accuracy and classification, particularly in the early years of the study. For example, the classification of JIA subtypes and periodic fever syndromes has been refined in recent years, possibly leading to misclassification in earlier cases. Third, treatment protocols were not standardized across the study period. Variability in therapeutic approaches, drug availability, and physician preferences could have affected both outcomes and complication rates, making cross-comparisons between subgroups more complex. Fourth, the single-center design may have introduced referral bias, potentially leading to underrepresentation of rarer rheumatic diseases that are either not referred to tertiary care or are misdiagnosed in primary settings. Some rare conditions such as interferonopathies, Blau syndrome, or localized scleroderma might be underreported for this reason. Finally, due to the diversity of diseases included, we focused primarily on overall disease outcomes (improvement versus no improvement) and could not provide detailed treatment data for each etiology. Given the single-center design of our study, the generalizability of our results could be limited. Despite these limitations, the study’s strengths include a large cohort, comprehensive inclusion of rare and common rheumatic causes, and valuable data from a region where certain conditions (e.g., FMF, IgAV) are highly prevalent but underreported globally.

## 5. Conclusion

This study provides a comprehensive overview of childhood-onset arthritis, encompassing both common etiologies such as JIA and regionally prevalent conditions like FMF and IgAV, as well as rarer autoimmune disorders. By integrating diverse causes within a large single-center cohort, our findings offer clinicians a practical and regionally relevant framework for diagnosis. Recognizing disease-specific patterns and considering local epidemiology are essential for accurate diagnosis and timely treatment. A multifaceted, context-aware diagnostic approach remains key to improving outcomes and ensuring a better quality of life for children affected by arthritis.

## Figures and Tables

**Figure:**
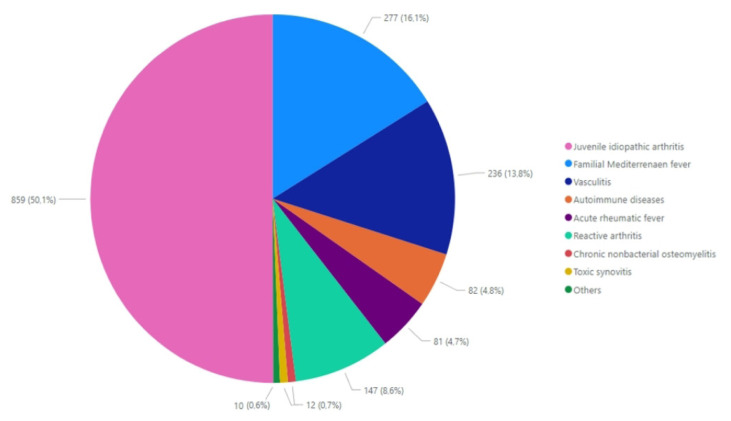
Rheumatic etiologies of pediatric patients presenting with arthritis.

**Table 1:** Demographic and clinical features of pediatric patients presenting with arthritis.

Diagnosis (number of patients)	Age at diagnosis, years, median (Q1–Q3)	Sex, female, n (%)	Clinical features except for arthritis, n (%)
Oligoarticular JIA (n = 396)	4.3 (2.9–6.8)	261 (65.9)	Uveitis (n = 36), IBD (n = 11)
Enthesitis-related arthritis (n = 195)	9.5 (6.7–12.8)	54 (27.7)	Sacroiliitis (n = 173), enthesitis (n = 43), uveitis (n = 4), IBD (n = 1)
Polyarticular JIA (n = 132)	6.6 (3.4–10.5)	72 (54.5)	Uveitis (n = 12), IBD (n = 4)
Systemic JIA (n = 107)	5.2 (1.9–6.7)	92 (50.3)	Fever (n = 107), skin rash (n = 91), lymphadenopathy (n = 61), hepatomegaly/splenomegaly (n = 43), serositis (n = 11), MAS (n = 37)
Psoriatic arthritis (n=20)	9.4 (6.9–12.8)	12 (60)	Psoriasis (n = 13)
Unclassified JIA (n=9)	8.4 (6.7–12.6)	5 (55.6)	Uveitis (n = 1)
Familial Mediterranean fever (n=277)	6.2 (4.3–8.7)	161 (58.1)	Fever (n = 255), abdominal pain (n = 204), chest pain (n = 71), ELE (n = 32)
Immunoglobulin A vasculitis (n = 166)	6.9 (4.7–9.5)	63 (37.9)	Skin rash (n = 166), GIS (n = 75) or renal (n = 31) involvement
Behcet’s disease (n = 27)	14.9 (12.2–16.3)	18 (66.7)	Oral aphtha (n = 27), genital ulcers (n = 10), skin rash (n = 9), uveitis (n = 7), thrombosis (n = 5), CNS involvement (n = 3)
Kawasaki disease (n = 18)	5.4 (4.3–7.8)	7 (38.9)	Skin rash (n = 16), conjunctivitis (n = 15), oromucosal changes (n = 14), lymphadenopathy (n = 13), edema or peeling in hands/feet (n = 7), coroner artery aneurism (n = 1)
Polyarteritis nodosa (n = 14)	6.7 (5.1–9.7)	6 (42.9)	Constitutional symptoms (n = 11), myalgia (n = 9), skin rash (n = 8), abdominal pain (n = 7), peripheral neuropathy (n = 2), GIS involvement (n = 1)
Adenosine deaminase 2 deficiency (n = 9)	5.8 (4.2–8.9)	5 (55.6)	Constitutional symptoms (n = 7), skin rash (n = 7), abdominal pain (n = 3), CNS (n = 3) or GIS (n = 1) involvement
Reactive arthritis (n = 147)	8.3 (5.2–12.6)	72 (48.9)	Skin rash (n = 21), conjunctivitis (n = 17), urethritis (n = 5)
Acute rheumatic fever (n = 81)	10.8 (7.4–13.7)	35 (43.2)	Carditis (n = 26), erythema marginatum (n = 14), subcutaneous nodules (n = 5), Sydenham chorea (n = 3)
Systemic lupus erythematosus (n = 53)	13.1 (10.8–14.9)	42 (79.2)	Skin rash (n = 44), photosensitivity (n = 42), oral aphtha (n = 23), hematologic findings (n = 20), nephritis (n = 12), CNS (n = 3) or GIS (n = 2) involvement
Juvenile dermatomyositis (n = 16)	6.8 (4.9–9.3)	7 (43.8)	Proximal muscle weakness (n = 16), skin rash (n = 15), calcinosis (n = 5), lung (n = 2) or GIS (n = 1) involvement
MCTD (n = 7)	13.4 (11.1–15.3)	7 (100)	Puffy hand (n = 5), oral aphtha (n = 5), skin rash (n = 4), GIS (n = 1) involvement
Rhupus syndrome (n = 5)	14.2 (12.2–16.3)	4 (80)	Skin rash (n = 5), photosensitivity (n = 4), oral aphtha (n = 3), hematologic findings (n = 1)
Toxic synovitis (n = 12)	2.8 (1.3–4.2)	7 (53.8)	Limping (n = 12)
Chronic nonbacterial osteomyelitis (n = 12)	10.5 (7.4–12.6)	5 (41.7)	Skin rash (n = 1), IBD (n = 1)
Others* (n = 10)	4.7 (3.6–8.1)	5 (50)	Constitutional symptoms (n = 4), skin rash (n = 4), abdominal pain (n = 1), lymphadenopathy (n = 1), conjunctivitis (n = 1), audiovestibular symptoms (n = 1), lung (n = 2) or CNS (n = 1) involvement

**Abbreviations:** CNS, central nervous system; ELE, erysipelas-like erythema; GIS, gastrointestinal system; IBD, inflammatory bowel disease; JIA, juvenile idiopathic arthritis; MAS, macrophage activation syndrome; MCTD, mixed connective tissue disease.

* Cryopyrin-associated periodic syndrome (n = 2), Blau syndrome (n = 2), type 1 interferonopathies (n = 2), mevalonate kinase deficiency (n = 1), localized scleroderma (n = 1), granulomatosis with polyangiitis (n = 1), Cogan syndrome (n = 1).

**Table 2:** Laboratory findings and outcomes of pediatric patients presenting with arthritis.

Diagnosis (number of patients)	Laboratory findings		Disease duration, years, median (Q1–Q3)	Outcome, n (%)	
	Elevated APR, n (%)	Specific tests		Improvement	No improvement
Oligoarticular JIA (n = 396)	213 (53.8)	ANA (+) (n = 282, 71.8%)	6.5 (2.8**–**9.1)	391 (98.7)	8 (1.3)
Enthesitis-related arthritis (n = 195)	174 (89.2)	HLA-B27 (+) (n = 101, 51.8%)	5.3 (3.1**–**7.6)	184 (94.4)	11 (5.6)
Polyarticular JIA (n = 132)	122 (92.4)	ANA (+) (n=48, 36.4%), RF (n = 27, 20.5%)	4.5 (2.3**–**5.9)	117 (88.6)	15 (11.4)
Systemic JIA (n = 107)	107 (100)	-	5.9 (1.7**–**8.3)	93 (86.9)	14 (13.1)
Psoriatic arthritis (n = 20)	18 (90)	ANA (+) (n = 12, 60%)	4.4 (2.2**–**5.8)	18 (80)	2 (10)
Unclassified JIA (n = 9)	8 (88.9)	ANA (+) (n = 2, 22.2%)	4.5 (2.8**–**5.7)	8 (88.9)	1 (11.1)
Familial Mediterranean fever (n = 277)	277 (100)	-	6.3 (4.1**–**9.6)	264 (95.3)	13 (4.7)
Immunoglobulin A vasculitis (n = 166)	151 (90.9)	-	4.2 (1.1**–**5.7)	161 (96.9)	5 (30.1)
Behcet’s disease (n = 27)	8 (29.6)	HLA-B51 (+) (n = 14, 51.9%)	6.8 (3.5**–**8.7)	23 (85.2)	4 (14.8)
Kawasaki disease (n = 18)	18 (100)	-	5.2 (1.8**–**7.5)	17 (94.4)	1 (5.6)
Polyarteritis nodosa (n = 14)	14 (100)	-	5.4 (3.1**–**8.3)	13 (92.9)	1 (7.1)
Adenosine deaminase 2 deficiency (n = 9)	9 (100)	-	3.5 (1.6**–**4.8)	7 (77.8)	2 (22.2)
Reactive arthritis (n = 147)	93 (63.2)	HLA-B27 (+) (n = 25/79, 31.6%)	2.3 (1.4**–**6.5)	147 (100)	0
Acute rheumatic fever (n = 81)	81 (100)	-	5.8 (2.5**–**9.3)	73 (90.1)	8 (9.9)
Systemic lupus erythematosus (n = 53)	49 (92.4, but only ESR)	ANA (+) (n=53, 100%), anti dsDNA (+) (n = 44, 83.1%), hypocomplementemia (n = 42,79.2%)	5.2 (3.6**–**7.8)	48 (90.6)	5 (9.4)
Juvenile dermatomyositis (n = 16)	12 (75)	ANA (+) (n = 7, 43.7%)	3.7 (2.5**–**5.4)	15 (93.7)	1 (6.3)
MCTD (n = 7)	5 (71.4)	ANA (+) (n = 7, 100%)	4.6 (2.1**–**6.3)	6 (85.7)	1 (14.3)
Rhupus syndrome (n = 5)	5 (100)	ANA (+) (n = 5, 100%)	2.2 (1.3**–**4.1)	4 (80)	1 (20)
Toxic synovitis (n = 12)	7 (58.3)	-	1.5 (0.9**–**2.7)	12 (100)	0
Chronic nonbacterial osteomyelitis (n = 12)	11 (91.7)	HLA-B27 (+) (n = 4/9, 44.4%)	2.1 (1.5**–**2.9)	10 (83.3)	2 (16.7)
Others* (n = 10)	6 (85.7)	ANCA (+) (n = 1, 14.3%)	3.9 (2.1**–**5.6)	8 (80)	2 (20)

**Abbreviations:** ANA, antinuclear antibody; ANCA, antineutrophil cytoplasmic antibody; anti-dsDNA, antidouble-stranded DNA; APR, acute phase reactants; HLA, human leukocyte antigen; IBD, inflammatory bowel disease; JIA, juvenile idiopathic arthritis; MCTD, mix connective tissue disease; RF, rheumatoid factor.

* Cryopyrin-associated periodic syndrome (n = 2), Blau syndrome (n = 2), type 1 interferonopathies (n = 2), mevalonate kinase deficiency (n = 1), localized scleroderma (n = 1), granulomatosis with polyangiitis (n = 1), Cogan syndrome (n = 1).

**Table 3:** Detailed arthritis features of different rheumatic etiologies

Diagnosis (number of patients)	Arthritis duration, days, median (Q1–Q3)	Arthritis type, n (%)	Type of dominant joint involved, n (%)	Presence of erosion -deformity, n (%)
Oligoarticular JIA (n = 396)	44 (42**–**51)	Oligoarthritis (n = 396)	Peripheral (98.9) and larger (97.3)	3/308 (0.9) −8 (2.1)
Enthesitis-related arthritis (n = 195)	46 (42**–**57)	Oligoarthritis (n = 57), polyarthritis (n = 11)	Axial (96.9) and larger (94.4)	11/193 (5.7)**–**9 (4.6)
Polyarticular JIA (n = 132)	43 (42**–**54)	Polyarthritis (n = 132)	Peripheral (95.5) and smaller (98.5)	14/121 (11.6)**–**16 (12.1)
Systemic JIA (n = 107)	42 (42**–**47)	Oligoarthritis (n = 26), polyarthritis (n = 81)	Peripheral (99.1) and smaller (76.6)	6/57 (10.5) −9 (8.4)
Psoriatic arthritis (n = 20)	49 (45**–**59)	Oligoarthritis (n = 7), polyarthritis (n = 13)	Peripheral (100) and smaller (95)	3/20 (15) −5 (25)
Unclassified JIA (n = 9)	51 (48**–**65)	Oligoarthritis (n = 4), polyarthritis (n = 5)	Peripheral (77.8) and smaller (55.6)	1/9 (11.1) −2 (22.2)
Familial Mediterranean fever (n = 277)	3 (3**–**5)	Oligoarthritis (n = 203, mostly monoarthritis), polyarthritis (n = 74)	Peripheral (94.6) and larger (72.9)	0–0
Immunoglobulin A vasculitis (n = 166)	2 (1**–**4)	Oligoarthritis (n = 161), polyarthritis (n = 5)	Peripheral (100) and larger (96.4)	0**–**0
Behcet’s disease (n = 27)	4 (3**–**7)	Oligoarthritis (n = 23), polyarthritis (n = 4)	Peripheral (70.4) and larger (85.2)	1/22 (4.5) −0
Kawasaki disease (n = 18)	3 (2**–**5)	Oligoarthritis (n = 16), polyarthritis (n = 2)	Peripheral (100) and larger (83.3)	0**–**0
Polyarteritis nodosa (n = 14)	5 (3**–**7)	Oligoarthritis (n = 13), polyarthritis (n = 1)	Peripheral (100) and larger (92.9)	0**–**1 (7.1)
Adenosine deaminase 2 deficiency (n = 9)	5 (3**–**9)	Oligoarthritis (n = 7), polyarthritis (n = 2)	Peripheral (100) and larger (77.8)	0**–**2 (40)
Reactive arthritis (n = 147)	2 (1**–**5)	Oligoarthritis (n = 147, mostly monoarthritis)	Peripheral (89.1) and larger (97.9)	0**–**0
Acute rheumatic fever (n = 81)	3 (2**–**6)	Oligoarthritis (n = 23), polyarthritis (n = 58)	Peripheral (100) and larger (70.4)	0**–**0
Systemic lupus erythematosus (n = 53)	5 (3**–**9)	Oligoarthritis (n = 21), polyarthritis (n = 32)	Peripheral (100) and smaller (60.4)	0–0
Juvenile dermatomyositis (n = 16)	4 (3**–**7)	Oligoarthritis (n = 16)	Peripheral (100) and larger (87.5)	0–0
MCTD (n = 7)	6 (4**–**9)	Oligoarthritis (n = 2), polyarthritis (n = 5)	Peripheral (100) and smaller (85.7)	0**–**1 (14.3)
Rhupus syndrome (n = 5)	48 (42**–**57)	Oligoarthritis (n = 1), polyarthritis (n = 4)	Peripheral (80) and smaller (60)	4 (80)**–**1 (20)
Toxic synovitis (n = 12)	5 (3**–**8)	Monoarthritis (n = 12)	Axial (100) and larger (100)	0–0
Chronic nonbacterial osteomyelitis (n = 12)	7 (5**–**12)	Oligoarthritis (n = 12)	Axial (91.7) and smaller (91.7)	0–0
Others^*^ (n = 10)	10 (7**–**18)	Oligoarthritis (n = 8), polyarthritis (n = 2)	Peripheral (100) and larger (80)	0**–**3 (30)

**Table 4:** Summary comparison of the most common rheumatic etiologies of childhood-onset arthritis

Etiology	Frequency (n, %)	Typical arthritis type	Duration of arthritis (median, days)	Clinical findings	Laboratory features	Treatment response (improvement, %)
Juvenile idiopathic arthritis (JIA)	859 (50.1%)	Oligo- or polyarthritis	~43–51	Subtype-specific (e.g. fever in sJIA, uveitis in oligo-JIA)	ANA+, RF+, HLA-B27+ (varies by subtype)	94.4%
Familial Mediterranean fever (FMF)	277 (16.2%)	Recurrent mono-/oligoarthritis	3 (3–5)	Fever, abdominal/chest pain, ELE	↑APR during attacks	95.3%
Vasculitis (e.g. IgAV)	236 (13.8%)	Oligoarthritis	2 (1–4)	Palpable purpura, GIS/renal involvement	↑APR	96.9% (IgAV)
Reactive arthritis	147 (8.6%)	Mono-/oligoarthritis	2 (1–5)	Skin findings, conjunctivitis, urethritis	HLA-B27+ (in ~32%)	100%
Acute rheumatic fever (ARF)	81 (4.7%)	Migratory polyarthritis	3 (2–6)	Carditis, skin findings	↑APR	90.1%
Autoimmune diseases (e.g. SLE, JDM, MCTD)	82 (4.8%)	Oligo- or polyarthritis	4–6	Rash, nephritis, myositis, etc.	ANA+, anti-dsDNA+, hypocomplementemia	~90%

**Abbreviations:** ANA, antinuclear antibody; anti-dsDNA, anti-double-stranded DNA; APR, acute phase reactants; ELE, erysipelas-like erythema; GIS, gastrointestinal system; HLA, human leukocyte antigen; IBD, inflammatory bowel disease; JIA, juvenile idiopathic arthritis; JDM, juvenile dermatomyositis; MCTD, mix connective tissue disease; RF, rheumatoid factor; SLE, systemic lupus erythematosus.
